# Early-phase insulin secretion during mixed-meal tolerance testing predicts β-cell function and secretory capacity in cystic fibrosis

**DOI:** 10.3389/fendo.2024.1340346

**Published:** 2024-02-20

**Authors:** Saba Sheikh, Darko Stefanovski, Marissa J. Kilberg, Denis Hadjiliadis, Ronald C. Rubenstein, Michael R. Rickels, Andrea Kelly

**Affiliations:** ^1^ Division of Pulmonary and Sleep Medicine, Children’s Hospital of Philadelphia and Department of Pediatrics, University of Pennsylvania Perelman School of Medicine, Philadelphia, PA, United States; ^2^ Department of Clinical Studies-New Bolton Center, University of Pennsylvania School of Veterinary Medicine, Kennett Square, PA, United States; ^3^ Division of Endocrinology and Diabetes, Children’s Hospital of Philadelphia and Department of Pediatrics, University of Pennsylvania Perelman School of Medicine, Philadelphia, PA, United States; ^4^ Division of Pulmonary and Critical Care Medicine, Department of Medicine, University of Pennsylvania Perelman School of Medicine, Philadelphia, PA, United States; ^5^ Division of Allergy and Pulmonary Medicine, Department of Pediatrics, Washington University School of Medicine, St. Louis, MO, United States; ^6^ Division of Endocrinology, Diabetes & Metabolism, Department of Medicine and Institute for Diabetes, Obesity & Metabolism, University of Pennsylvania Perelman School of Medicine, Philadelphia, PA, United States

**Keywords:** insulin secretion, beta-cell function, beta-cell secretory capacity, cystic fibrosis, pancreatic insufficiency, cystic fibrosis-related diabetes

## Abstract

Insulin secretion within 30 minutes of nutrient ingestion is reduced in people with cystic fibrosis (PwCF) and pancreatic insufficiency and declines with worsening glucose tolerance. The glucose potentiated arginine (GPA) test is validated for quantifying β-cell secretory capacity as an estimate of functional β-cell mass but requires technical expertise and is burdensome. This study sought to compare insulin secretion during mixed-meal tolerance testing (MMTT) to GPA-derived parameters in PwCF.

**Methods:**

Secondary data analysis of CF-focused prospective studies was performed in PwCF categorized as 1) pancreatic insufficient [PI-CF] or 2) pancreatic sufficient [PS-CF] and in 3) non-CF controls. MMTT: insulin secretory rates (ISR) were derived by parametric deconvolution using 2-compartment model of C-peptide kinetics, and incremental area under the curve (AUC) was calculated for 30, 60 and 180-minutes. GPA: acute insulin (AIR) and C-peptide responses (ACR) were calculated as average post-arginine insulin or C-peptide response minus pre-arginine insulin or C-peptide under fasting (AIR_arg_ and ACR_arg_), ~230 mg/dL (AIR_pot_ and ACR_pot_), and ~340 mg/dL (AIR_max_ and ACR_max_) hyperglycemic clamp conditions. Relationships of MMTT to GPA parameters were derived using Pearson’s correlation coefficient. Predicted values were generated for MMTT ISR and compared to GPA parameters using Bland Altman analysis to assess degree of concordance.

**Results:**

85 PwCF (45 female; 75 PI-CF and 10 PS-CF) median (range) age 23 (6-56) years with BMI 23 (13-34) kg/m^2^, HbA_1c_ 5.5 (3.8-10.2)%, and FEV1%-predicted 88 (26-125) and 4 non-CF controls of similar age and BMI were included. ISR AUC_30min_ positively correlated with AIR_arg_ (*r*=0.55), AIR_pot_ (*r*=0.62), and AIR_max_ (*r*=0.46) and with ACR_arg_ (*r*=0.59), ACR_pot_ (*r*=0.60), and ACR_max_ (*r*=0.51) (all *P*<0.001). ISR AUC_30min_ strongly predicted AIR_arg_ (concordance=0.86), AIR_pot_ (concordance=0.89), and AIR_max_ (concordance=0.76) at lower mean GPA values, but underestimated AIR_arg_, AIR_pot_, and AIR_max_ at higher GPA-defined β-cell secretory capacity. Between test agreement was unaltered by adjustment for study group, OGTT glucose category, and BMI.

**Conclusion:**

Early-phase insulin secretion during MMTT can accurately predict GPA-derived measures of β-cell function and secretory capacity when functional β-cell mass is reduced. These data can inform future multicenter studies requiring reliable, standardized, and technically feasible testing mechanisms to quantify β-cell function and secretory capacity.

## Introduction

Medical advancements and improved clinical care in people with cystic fibrosis (PwCF) have improved life expectancy, with the majority of PwCF living well into adulthood (1). With advancing age, however, PwCF are at an increased risk for the development of cystic fibrosis-related diabetes (CFRD) (2), a major comorbidity that affects 40-50% of adults living with CF (2). Better understanding the pathophysiology and risk factors for diabetes development in PwCF is imperative to prevent, treat and manage CFRD.

While the underpinnings of CFRD development remain elusive, impaired insulin secretion is considered the primary defect, and the American Diabetes Association classifies CFRD as “specific types of diabetes due to other causes” (3). Individuals with exocrine pancreatic insufficient CF (PI-CF) are at greater risk for CFRD than PwCF with pancreatic sufficiency (4). While insulin secretion is largely preserved in pancreatic sufficient CF (PS-CF), it is decreased in PI-CF even in individuals with “normal” glucose tolerance (5). With worsening glucose tolerance in PI-CF, insulin and C-peptide peak concentrations are lower and their secretion is progressively delayed after nutrient ingestion; these findings indicate worsening impairment of early-phase insulin secretion dynamics (6). In addition to fibrofatty replacement of the exocrine pancreas in PI-CF, loss of CFTR function, genetic susceptibility, systemic and local inflammation, and disturbances in the entero-insular axis also appear to have roles in pancreatic β-cell dysfunction in CF (7, 8).

Reproducible and more easily available techniques that estimate β-cell function are required to advance our understanding of the development of insulin secretion defects in CF. While multiple methodologies exist to quantify β-cell function, each has their own limitations due to varying routes of administration, stimuli and sampling (9). We have successfully used the glucose potentiated arginine (GPA) test, which provides validated measures of functional β-cell mass, across multiple studies to evaluate β-cell function and secretory capacity in PwCF (5, 6, 10–12). β-cell responses at basal (fasting) glucose and under hyperglycemic clamp conditions are calculated in response to arginine, a non-glucose insulin secretagogue. However, this test requires technical expertise and resources that are not readily available at many research centers and is burdensome to participants. The mixed meal tolerance test (MMTT), which is less intensive in terms of participation and resources, can assess insulin secretion from mathematical deconvolution of peripherally measured C-peptide concentrations in response to a more physiologic challenge, a standardized meal (13).

In this study, we aimed to test the correlation of insulin secretory rate (ISR) responses during MMTT and GPA-derived acute insulin and C-peptide responses under fasting and hyperglycemic clamp conditions. Based on known CF pathophysiology (6) we hypothesized that ISR responses during the initial 30 minutes of the MMTT would correlate closely with and accurately predict GPA measures of β-cell function and secretory capacity, and also explored this relationship for ISR responses during the initial 60 minutes of the MMTT.

## Materials and methods

We performed these secondary analyses on data collected from PwCF who participated in research studies conducted between March 2012 and February 2022 at the Hospital of the University of Pennsylvania and the Children’s Hospital of Philadelphia and that included assessment of 1) β-cell function and secretory capacity by GPA and 2) glucose tolerance and insulin secretion by MMTT. Subjects ≥6 years of age with a confirmed clinical diagnosis of CF as per Cystic Fibrosis Foundation diagnostic criteria were included (14). Data from non-CF control participants from a study on the effects of incretin hormones on islet function in PI-CF were also included for comparison (12).

All participants completed a 75-gram oral glucose tolerance test (OGTT) within the previous 6 months and were characterized as having either normal glucose tolerance (NGT), defined as 1-hour glucose < 155 mg/dL and 2-hour glucose < 140 mg/dL, early glucose intolerance (EGI) defined as 1-hour glucose ≥ 155 mg/dL and 2-hour glucose < 140 mg/dL (6), impaired glucose tolerance (IGT) defined as 2-hour glucose ≥ 140 mg/dL and < 200 mg/dL), or CFRD defined as 2-hour glucose ≥ 200 mg/dL or previously confirmed diagnosis with or without fasting hyperglycemia (fasting glucose ≥ 126 mg/dL).

Participants were excluded if they had experienced clinically symptomatic pancreatitis within the previous 12 months, a history of lung or liver transplant, significant kidney or liver dysfunction, or were pregnant or nursing females. Study visits were delayed for acute illness necessitating change of antibiotics or treatment with oral or intravenous glucocorticoids within the prior 4 weeks. Approvals for each study were obtained through the institutional review boards of the University of Pennsylvania and the Children’s Hospital of Philadelphia. All adult participants or parents of children (age <18 years) provided written informed consent to participate and to use their deidentified data for future analyses; assent was provided by participants aged <18 years.

### Mixed meal tolerance test

A standardized MMTT was used to evaluate postprandial glucose tolerance and incretin and islet hormone secretion, as previously described (5). After a 12-hour overnight fast, an antecubital or forearm vein catheter was placed for blood sampling, with the arm warmed using a heating pad to promote arterialization of venous blood. After approximately 20 min of acclimatization to the catheter, baseline blood samples were taken at *t* = −10 and −1 min before consumption of an 820-kcal meal over 15 min starting at *t* = 0. Meal composition was 47% carbohydrate, 40% fat, and 13% protein of the total energy content (15). Pancreatic insufficient participants took their regularly prescribed dose of pancreatic enzyme replacement with the test meal. Additional blood samples were collected at *t* = 10, 15, 20, 30, 60, 90, 120, 150, 180, 210, and 240 min from the start of the meal. If applicable, rapid-acting insulin or repaglinide was held for 12 h prior to testing, while morning doses of CFTR modulator therapy were taken as prescribed with the meal.

### Glucose potentiated arginine test

A GPA test was performed according to established methodology for evaluation of β-cell function and secretory capacity (16–18). After a 12-hour overnight fast, an antecubital or forearm vein catheter was placed for infusions and a second catheter was placed in the contralateral forearm or hand for blood sampling, with the forearm or hand warmed using a heating pad to promote arterialization of venous blood. After approximately 20 min of acclimatization to placement of the catheters, baseline blood samples were taken at *t* = −5 and −1 min relative to injection of 10% arginine (5 g) over 1 min starting at *t* = 0. Additional blood samples were collected at *t* = 2, 3, 4, and 5 min. Beginning at *t* = 10 min, a hyperglycemic clamp technique (19) using a variable rate infusion of 20% dextrose was performed to achieve a plasma glucose concentration of ~230 mg/dL. Blood samples were taken every 5 min to adjust the glucose infusion rate and achieve the desired plasma glucose concentration. After 45 min of glucose infusion (at *t* = 55 min), a second arginine pulse was injected with identical blood sampling. The first administration of arginine has no effect on the subsequent response to arginine using this protocol (20). A subsequent 2-hour period without glucose infusion allowed plasma glucose to return to baseline. A second hyperglycemic clamp was then performed to achieve a plasma glucose concentration of ~340 mg/dL. After 45 min of glucose infusion, a third arginine pulse was injected with identical sampling. If applicable, rapid-acting insulin or repaglinide was held for 12 h prior to testing, and morning doses of CFTR modulator therapy were held until the completion of testing and taken with a provided meal.

### Biochemical analyses

Plasma glucose was measured in duplicate by the glucose oxidase method using an automated glucose analyzer (YSI 2300; Yellow Springs Instruments, Yellow Springs, OH, USA). Additional blood samples were collected into tubes on ice containing ethylenediamine tetra-acetate and protease inhibitor cocktail and, for the MMTT test, dipeptidyl peptidase-4 inhibitor (Sigma-Aldrich, St. Louis, MO, USA). Samples were centrifuged at 4°C, separated, and frozen at −80°C for subsequent analysis. Plasma insulin (Millipore Cat# HI-14 K, RRID: AB_2801577) and C-peptide (Millipore Cat# HCP-20 K, RRID: AB_2891151) were assayed in duplicate by double-antibody radioimmunoassay (Millipore, Billerica, MA, USA).

### Calculations

ISRs during the MMTT were calculated from C-peptide values and derived by parametric deconvolution of C-peptide kinetics using a 2-compartment model (21) in WinSAAM software 3.0.8 (University of Pennsylvania, New Bolton Center, Kennett Square, PA, US). The 30-, 60- and 180-min incremental areas under the curve for ISR (ISR AUC_30min_, ISR AUC_60min_, ISR AUC_180min_, respectively) were calculated with baseline values subtracted using the trapezoidal method in STATA 15 software (StataCorp LP, College Station, TX, USA). Acute insulin and C-peptide responses to arginine (AIR_arg_ and ACR_arg_, respectively) during the GPA test were calculated as mean of 2-, 3-, 4-, and 5-min values minus mean of baseline values (-5 and -1 min) (19). Acute responses during the 230 mg/dL clamp enable determination of glucose potentiation of arginine-induced insulin (AIR_pot_) and C-peptide (ACR_pot_) release. Acute responses during the 340 mg/dL clamp allow for determination of maximum arginine-induced insulin (AIR_max_) and C-peptide (ACR_max_) release (i.e., β-cell secretory capacity).

### Statistical analyses

Data are reported as median (min-max) and categorial data as percentages, except where indicated. Graphs were generated to permit visual inspection of data by study group: PI-CF, PS-CF, and non-CF controls. Subject demographics were examined using the Kruskal-Wallis test for continuous variables and χ2 test for categorical variables. Comparison of results between the PI-CF, PS-CF, and control subjects was performed with the Kruskal-Wallis test, and when significant differences at *P* ≤ 0.05 (two-tailed) were found, comparisons between groups were performed using the Dunn test. Comparison of results across glucose tolerance categories among PI-CF was performed with the nonparametric test for progressively worsening differences across ordered groups (*nptrend*).

Pearson’s correlation coefficient was used to assess the relationship between ISR AUC during MMTT to acute insulin and C-peptide responses during the GPA. As the MMTT and GPA measures are with different units and not directly comparable through a Bland Altman analysis, we corrected the ISR AUCs using linear regression to generate predicted values from MMTT to compare to GPA measures. These models were generated with MMTT measures as the independent variable and GPA measures as the outcome variable. GPA values were compared to predicted values from MMTT using the *concord* command in STATA that generates the Bland Altman Analysis, along with a Pearson’s correlation coefficient, *r*, the bias-correction factor, concordance (measure of accuracy), and Lin’s correlation coefficient, rho_c (product of Pearson’s *r* and concordance). Linear models were also adjusted for age, sex, study group, OGTT glucose category and BMI to evaluate if the between test agreement was modified by these factors. As age and sex were not significantly associated with our outcome variable they were excluded from the final model. All data analyses were performed in STATA 15 software.

## Results

### Participants

A total of 89 CF participants and 5 non-CF controls were enrolled in studies that included baseline assessment of both MMTT and GPA during an approximate 10-year period (5, 6, 10–12). Four CF participants and one non-CF control were unable to complete one or both tests and were, therefore, excluded from the present analyses. Data were subsequently analyzed for 85 CF participants and 4 non-CF controls. The majority of CF participants were pancreatic insufficient (PI-CF; *n* = 75) and were more often homozygous (46%) or heterozygous (37%) for the 508del *CFTR* mutation (*P* = 0.02) and glucose intolerant by OGTT (*P* = 0.002) than the group with pancreatic sufficient CF (PS-CF; *n* = 10; **Table 1**). HbA_1c_ was higher in PI-CF than PS-CF whose HbA_1c_ was not different than controls (PI-CF vs PS-CF: *P* = 0.001; **Table 1**). Only 29 CF participants (34%) were on CFTR modulator therapy at the time of their metabolic testing visits, of whom 16 were on highly effective modulator therapy.

**Table 1:** Subject characteristics.

	PI-CF (*n* = 75)	PS-CF (*n* = 10)	Non-CF Controls (*n* = 4)	*P-value*
**Female, n (%)**	39 (52)	6 (60)	2 (50)	0.90
**Age, years**	22 (6.1-50.0)	30.5 (14.1-56.0)	28 (19-30)	0.12
**Weight-Z**	0.0 (-4.3-2.2)	0.5 (-0.9-2.3)	0.2 (-0.8-1.6)	0.41
**Height-Z**	-0.1 (-3.0-2.0)	0.2 (-2.1-0.9)	0.1 (-1.0-1.9)	0.74
**BMI-Z**	0.1 (-2.3-1.9)	0.6 (0.7-2.0)	0.1 (-0.4-1.1)	0.41
**BMI, kg/m^2^**	23 (13-34)	24 (19-33)	23 (21-27)	0.28
**FEV_1_pp**	88 (27-125)	91 (26-112)	N/D	0.96
**CFTR mutations** **- F508Del Homozygote** **- F508Del Heterozygote** **- Other**	34 (46) 28 (37) 13 (17)	0 (0) 6 (60) 4 (40)	N/A	0.02
**HbA_1c_, %**	5.6 (4.7-10.2)	5.2 (3.8-5.7)	5.2 (5.2-5.3)	0.001
OGTT classification* - **NGT** - **EGI** - **IGT** - **Diabetes**	18 (24) 28 (37) 16 (21) 11 (15)	7 (70) 2 (20) 0 (0) 0 (0)	4 (100) 0 (0) 0 (0) 0 (0)	0.002
**Fasting blood glucose^, mg/dL**	90 (64-139)	92 (74-102)	88 (85-90)	0.84
**1-hr OGTT blood glucose^#^, mg/dL**	180 (91-399)	118 (67-165)	139 (121-141)	0.0003
**2-hr OGTT blood glucose^+^, mg/dL**	113 (34-336)	102 (46-115)	91 (87-105)	0.07
**CFTR modulator use** - **Ivacaftor** - **Lumacafotor/Ivacaftor** - **Tezacaftor/Ivacaftor** - **Elexacaftor/Tezacaftor/Ivacaftor** - **None**	5 (7) 5 (7) 1 (1) 10 (13) 54 (72)	1 (10) 0 (0) 0 (0) 0 (0) 9 (90)	N/A	0.42

Data are medians and ranges (min–max) for continuous variables and number and percentage for categorical variables. CFTR, cystic fibrosis transmembrane regulator; EGI, early glucose intolerance (1-hour glucose ≥ 155 mg/dL & 2-hour glucose < 140 mg/dL); FEV1pp, percent predicted forced expiratory volume in 1 second; HbA_1c_, glycosylated hemoglobin; IGT, impaired glucose tolerance (2-hour glucose ≥ 140 mg/dL & < 200 mg/dL); N/A, not applicable; NGT, normal glucose tolerance (1-hour glucose < 155 mg/dL & 2-hour glucose < 140 mg/dL); OGTT, oral glucose tolerance test; PI-CF, pancreatic insufficient cystic fibrosis; PS-CF, pancreatic sufficient cystic fibrosis.

* Classification based on Nyirjesy et al. (6); OGTT missing for 2 PI-CF and 1 PS-CF.

^ Fasting blood glucose not available for 8 subjects.

^#^1-hr OGTT blood glucose not available for 9 subjects.

^+^ 2-hr OGTT blood glucose not available for 9 subjects.

### β-cell function with MMTT

During the first 30- and 60-min following meal ingestion, PI-CF subjects had lower ISR AUC_30 min_ and ISR AUC_60 min_ compared to PS-CF and non-CF controls (*P* < 0.05 for all comparisons; **Table 2**). In contrast, ISR AUC_180 min_ was not different across groups.

**Table 2:** β-cell function measures derived from the mixed-meal tolerance and glucose-potentiated arginine tests.

	PI-CF (*n* = 75)	PS-CF (*n* = 10)	Controls (*n* = 4)	Overall *P-value*		
				PI-CF vs PS-CF	PI-CF vs controls	PS-CF vs controls
ISR AUC_30 min,_ μIU/mL	99^*^ (0-593)	307 (99-640)	264 (140-453)	0.0004	0.0005 0.01	0.49
ISR AUC_60 min,_ μIU/mL	368^*^ (17-1352)	821 (392-1236)	791 (429-1001)	0.0005	0.0009 0.02	0.47
ISR AUC_180 min,_ μIU/mL	1509^*^ (309-5097)	1765 (1110-2320)	2216^^^ (1502-3008)		0.15	
AIR_arg_, μU/mL	14.7^*^ (0-70.3)	37.0 (16.1-59.8)	35.7 (32.5-50.6)	0.0001	0.0001 0.001	0.32
AIR_pot_, μU/mL	44.2^*^ (5.8-392.3)	150.0 (78.0-337.7)	97.7 (270.7-392.2)	<0.001	0.0001 0.008	0.35
AIR_max_, μU/mL	54.9^#^ (13.4-398.4)	113.1 (69-605.7)	79.5^^^ (60.9-371.6)	0.0007	0.003 0.07	0.36
ACR_arg_, ng/mL	0.8 (0.1-3.9)	1.7 (0.7-2.9)	2.2 (2.1-3.5)	0.0001	0.0001 0.0003	0.20
ACR_pot_, ng/mL	2.3^+^ (0.2-9.2)	5.7 (3.1-10.6)	5.0 (4.7-13.9)	<0.001	0.0001 0.002	0.39
ACR_max_, ng/mL	2.6^+^ (0.0-9.9)	4.9 (2.4-13.2)	3.5^^^ (2.3-9.7)	0.003	0.01 0.14	0.33

ACR_arg_, acute C-peptide responses to arginine; ACR_pot_, acute C-peptide responses to glucose-potentiated arginine; ACR_max_, acute C-peptide responses to maximally glucose-potentiated arginine; AIR_arg_, acute insulin responses to arginine; AIR_pot_, acute insulin responses to glucose-potentiated arginine; AIR_max_, acute insulin responses to maximally glucose-potentiated arginine; ISR AUC, incremental area under curve for insulin secretory rate.

Data are medians and ranges (min–max). Between-group comparisons performed when overall P-value was significant at P ≤ 0.05 (two-tailed).

^*^n = 73 for PI-CF.

^^^n = 3 for controls.

^#^n = 71 for PI-CF.

^+^n = 74 for PI-CF.

Within the PI-CF subjects, ISR AUC_30 min_ and ISR AUC_60 min_ were progressively worse with progressive impairment in glucose tolerance from NGT to CFRD (*P* = 0.01 and *P* = 0.04, respectively; data not shown), confirming previous results from a smaller cohort of 42 individuals with PI-CF (6). No differences were found in ISR AUC_180 min_ across glucose tolerance categories within PI-CF subjects.

### β-cell function and secretory capacity with GPA

During the GPA test, PI-CF subjects had lower AIR_arg_ and AIR_pot_ compared to PS-CF and controls (*P* < 0.05 for all comparisons; **Table 2**). AIR_max_ was lower in PI-CF than PS-CF (*P* = 0.001), but was only lower by trend in PI-CF compared to the smaller sample of non-CF controls (*P* = 0.14). No differences were observed in acute insulin responses between PS-CF and non-CF controls. Similar between group results were found for acute C-peptide responses at fasting (ACR_arg_) and under hyperglycemic clamp conditions (ACR_pot_ and ACR_max_; **Table 2**).

Across glucose tolerance categories in PI-CF, while AIR_arg_ trended lower (*P* = 0.07), AIR_pot_ and AIR_max_ were progressively lower with worsening glucose tolerance (*P* = 0.001 & 0.01, respectively; data not shown). Similar results were seen for acute C-peptide responses across glucose tolerance categories in PI-CF.

### Comparison of β-cell function measures between MMTT and GPA

**Figure 1:**
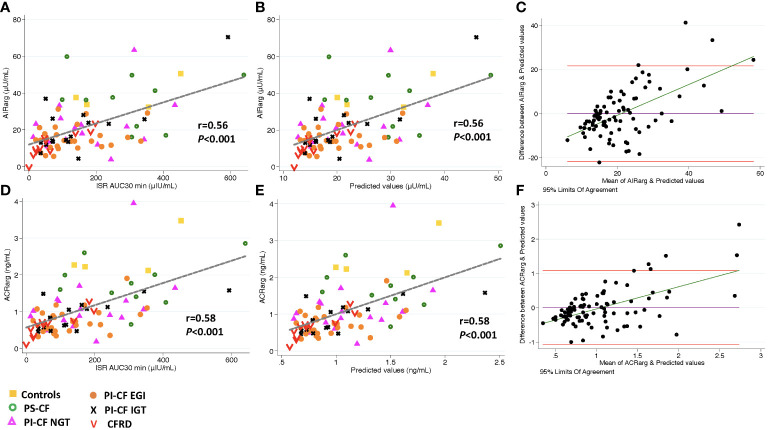
There is a positive correlation between AIR_arg_, and ISR AUC_30 min_ [incremental area under curve for ISR at 30 min; **(A)**], and predicted values from linear regression modeling [**(B)**, AIR_arg_=β ^*^ISR iAUC_30 min_ intercept]. Bland Altman plot shows increasing bias between between GPA estimated AIR_arg_ & MMTT predicted values as mean values increase **(C)**. Horizontal red lines depict 95% limits of agreement and green line depicts the bias. **(D–F)** demonstrate similar relationships for ACR_arg_.

**Figure 2:**
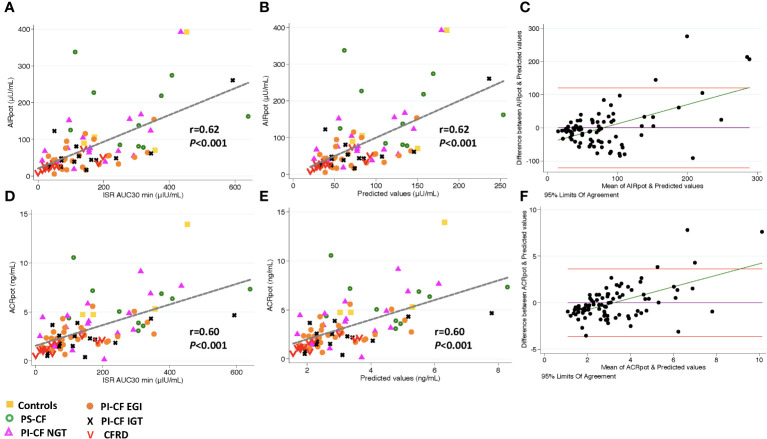
There is a positive correlation between AIR_pot_ and ISR AUC_30 min_ [incremental area under curve for ISR at 30 min; **(A)**], and predicted values from linear regression modeling [**(B)**, AIR_pot_=β^*^ ISR iAUC_30 min_ + intercept]. Bland Altman plot shows increasing bias between between GPA estimated AIR_pot_ & MMTT predicted values as the mean values increase **(C)**. Horizontal red lines depict 95% limits of agreement and green line depicts the bias. **(D–F)** demonstrate similar relationships for ACR_pot_.

**Figure 3:**
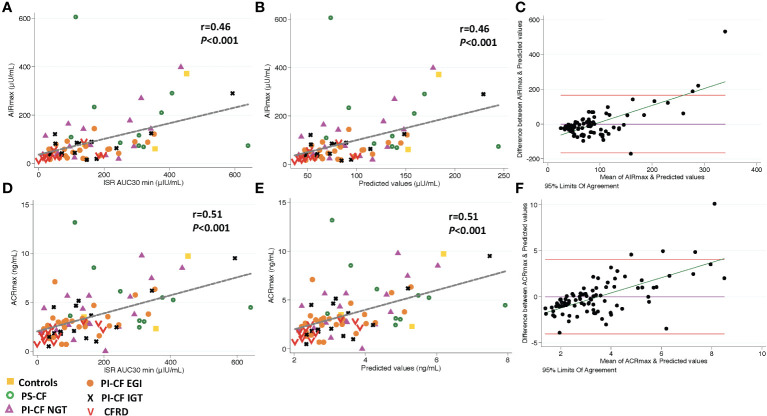
There is a positive correlation between AIR_max_ and and ISR AUC_30 min_ [incremental area under curve for ISR at 30 min; **(A)**], and predicted values from linear regression modeling [**(B)**, AIR_max_=β^*^ ISR ¡AUC_30 min_ intercept]. Bland Altman plot shows increasing bias between between GPA estimated AIR_max_ & MMTT predicted values as the mean values increase **(C)**. Horizontal red lines depict 95% limits of agreement and green line depicts the bias. **(D–F)** demonstrate similar relationships for ACR_max_.

The ISR AUC_30min_ was positively correlated with AIR_arg_, AIR_pot_ and AIR_max_ (**Figures 1A**, **2A**, **3A**; **Table 3**) as well as with ACR_arg_, ACR_pot_ and ACR_max_ (**Figures 1D**, **2D**, **3D**; **Table 3**).

**Table 3:** Relationship of mixed-meal tolerance test measures of early-phase insulin secretion to glucose-potentiated arginine test measures of acute insulin and C-peptide responses.

	ISR AUC_30 min_			ISR AUC_60 min_		
	Pearson’s r	Spearman’s rho	Concordance	Pearson’s r	Spearman’s rho	Concordance
AIR_arg_, μU/mL	0.56	0.49	0.86	0.54	0.49	0.84
AIR_pot_, μU/mL	0.62	0.61	0.89	0.52	0.64	0.82
AIR_max_, μU/mL	0.46	0.48	0.76	0.38	0.50	0.67
ACR_arg_, ng/mL	0.59	0.57	0.87	0.56	0.55	0.85
ACR_pot_, ng/mL	0.60	0.60	0.89	0.53	0.59	0.83
ACR_max_, ng/mL	0.51	0.53	0.81	0.47	0.50	0.76

ACR_arg_, acute C-peptide responses to arginine; ACR_pot_, acute C-peptide responses to glucose-potentiated arginine; ACR_max_, acute C-peptide responses to maximally glucose-potentiated arginine; AIR_arg_, acute insulin responses to arginine; AIR_pot_, acute insulin responses to glucose-potentiated arginine; AIR_max_, acute insulin responses to maximally glucose-potentiated arginine; ISR AUC, incremental area under curve for insulin secretory rate.

Model predicted values from ISR AUC_30 min_ were positively correlated to AIR_arg_ (Pearson’s *r* = 0.56, *P* < 0.001, **Figure 1B**) and accurately able to predict AIR_arg_ (concordance = 0.86, **Table 3**). Bland Altman analysis showed acceptable bias at lower mean AIR_arg_ values, but as mean values increase, the bias also increases (**Figure 1C**). When regression models were adjusted for study group, OGTT-defined glucose tolerance category, and BMI, the results were similar, suggesting that pancreatic sufficiency status, glucose tolerance and BMI do not affect MMTT performance. Similarly, the positive and predictive relationship between ISR AUC_30 min_ and ACR_arg_ also demonstrates greater bias as mean values of ACR_arg_ increase (**Figures 1E, F**, **Table 3**).

Model predicted values from ISR AUC_30 min_ were more strongly positively correlated with AIR_pot_ (*r* = 0.62; *P*< 0.001; **Figure 2B**) and very accurately predicted AIR_pot_ (concordance = 0.89, **Table 3**). Bland Altman analysis again showed acceptable bias at lower mean AIR_pot_ and increasing bias as mean values increase (**Figure 2C**). When regression models were adjusted for study group, OGTT-defined glucose tolerance category, and BMI, the results were similar. Results were also similar for the positive and predictive relationship between ISR AUC_30 min_ and ACR_pot_, showing increasing bias with increases in ACR_pot_ (**Figures 2E, F**; **Table 3**).

Similar to AIR_arg_ and AIR_pot_, model predicted values from ISR AUC_30 min_ were positively correlated with AIR_max_ (*r* = 0.46; *P <*0.001; **Figure 3B**) and accurately predicted AIR_max_ (concordance = 0.76; **Table 3**). Bland Altman analysis again showed acceptable bias at lower mean AIR_pot_ that increased as mean values increased (**Figure 3C**). When regression models were adjusted for study group, OGTT-defined glucose tolerance category, and BMI, the results were similar. Results were also similar for the positive and predictive relationship between ISR AUC_30 min_ and ACR_max_ (**Figures 3E, F**; **Table 3**).

**Figure 4:**
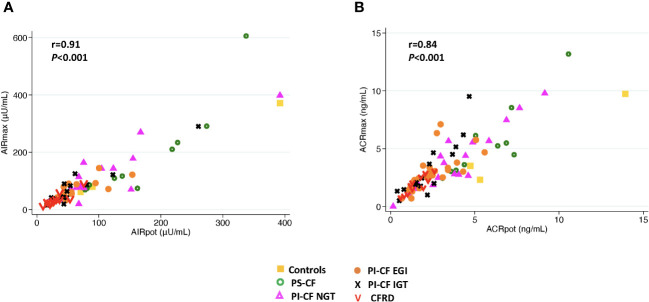
There is a positive correlation between AIR_max_ and AIR_pot_ **(A)** and between ACR_max_ and ACR_pot_ **(B)**.

When ISR AUC_60 min_ was explored to model predicted values for acute insulin and C-peptide responses, very similar results were obtained for Pearson’s correlation and concordance (**Table 3**). In addition, AIR_pot_ was highly correlated with AIR_max_ (*r* = 0.91; *P*< 0.001; **Figure 4A**), with a similar strong relationship between ACR_pot_ and ACR_max_ (*r* = 0.84; *P*< 0.001; **Figure 4B**).

## Discussion

These analyses tested the agreement between measures of insulin secretion derived from a MMTT, which estimates β-cell function in response to an oral challenge, and those derived from the GPA test, which estimates β-cell secretory capacity (or functional β-cell mass), in PwCF. Our data suggest that early-phase insulin secretion as defined by the ISR AUC_30 min_ during MMTT demonstrates good agreement with β-cell secretory capacity measured from glucose-potentiation of arginine-induced insulin secretion. However, in subjects with high β-cell secretory capacity, the MMTT measure of early-phase insulin secretion is biased toward underestimating the β-cell secretory capacity. These findings suggest that early-phase insulin secretion during MMTT peaks before exhausting the reserve capacity of the β-cells for insulin secretion. Therefore, while the MMTT may not adequately assess functional β-cell mass in a fully functional pancreas, it does provide a close estimate of β-cell secretory capacity in people who have a reduced functional β-cell mass, such as PwCF and abnormal glucose tolerance (6).

β-cell secretory capacity provides the best estimate of functional β-cell mass and is measured from glucose-potentiation of insulin or C-peptide release in response to a non-glucose secretagogue, such as arginine (22). In advanced type 2 diabetes (T2D), β-cell secretory capacity is diminished without a change in β-cell sensitivity to glucose (23, 24), while early in T2D impaired β-cell sensitivity to glucose is present with relative preservation of the reserve capacity for insulin secretion that is evident with maximal glucose-potentiation (25). In contrast, we have demonstrated a decrease in β-cell secretory capacity in PI-CF subjects that progresses with worsening glucose tolerance status without any impairment in β-cell sensitivity to glucose (5, 6). These differences may be explained by a more significant defect in β-cell function than mass early in T2D, with decreased β-cell sensitivity to glucose possibly representing a protective mechanism against the potential for β-cell exhaustion induced by chronically increased demand for secretion. In contrast, the primary defect in PI-CF appears to be a reduced β-cell secretory capacity (5) which may result from a combination of reduced β-cell numbers as well as reduced ability of residual β-cells to manufacture and release insulin. The reduced β-cell secretory capacity without impairment in β-cell sensitivity to glucose observed in PI-CF has also been demonstrated in other conditions with reduced functional β-cell mass including early type 1 diabetes (T1D) (26) and islet transplant recipients (27). Importantly, in the absence of impaired β-cell sensitivity to glucose, glucose-potentiation at ~230 mg/dL is sufficient for estimation of the β-cell secretory capacity from AIR_pot_ or ACR_pot_ and can obviate the need for a second, ~340 mg/dL hyperglycemic clamp to obtain maximal responses AIR_max_ or ACR_max_ (28), as demonstrated in the present report for PwCF.

The main limitations to performing GPA routinely in the research setting are the technical expertise requirement and high participant burden. Both an OGTT and MMTT are easier to perform at most research centers, are standardized, and consistency can be maintained across studies. The MMTT is more physiologic than an OGTT, hence subjects experience fewer adverse effects including nausea and hypoglycemia. The MMTT also allows robust interrogation of the entero-insular axis in the stimulation of insulin secretion. Unlike GPA (and OGTT), response measures during a MMTT are affected by the degree of nutrient digestion and absorption and neurohormonal responses to nutrient ingestion including the entero-insular axis. Nutrient absorption in CF is known to be impaired in the presence of exocrine pancreatic insufficiency which is present in ~85% of PwCF (1). Furthermore, digestive problems related to pancreatic insufficiency, such as rate of gastric emptying, also impair secretion of the incretin hormones, glucagon-like peptide-1 (GLP-1) and glucose-dependent insulinotropic polypeptide (GIP), from intestinal L and K cells, respectively (29, 30). GLP-1 improves postprandial glycemia through slowing gastric emptying and glucose-dependent insulinotropic and glucagonostatic properties. GIP is insulinotropic, and can stimulate glucagon secretion (31). However, this entero-insular axis is impaired in PwCF (30, 32). The use of MMTT, rather than an OGTT, allows for more complete examination of this axis.

Diminished incretin secretion in CF may contribute to the aberrant post-prandial insulin secretion and glucose homeostasis and likely contributes to the pathophysiology of diabetes in CF (5). Pancreatic enzyme replacement therapy (PERT) improves but does not normalize GLP-1 and GIP secretion (5, 32). Inhibitors of dipeptidyl peptidase-4 (DPP-4) prevent inactivation of endogenous GLP-1 and GIP, and our group has shown that treatment of adults with PI-CF and abnormal glucose tolerance with the DPP-4 inhibitor sitagliptin for 6 months improved the rate and degree of postprandial insulin secretion during the MMTT, although did not improve post-prandial glucose tolerance (11). This favorable effect upon insulin secretion may be attributed to GLP-1 as infusion of GLP-1, but not of GIP, improves glucose-dependent β-cell insulin secretion in PI-CF (12).

The data here also demonstrate higher GPA-derived acute insulin and C-peptide responses in non-CF controls and PS-CF compared to PI-CF. While agreement between MMTT-derived ISR AUC_30 min_ appears weaker with higher GPA measures, linear regression models did not demonstrate differences in agreement when adjusted for study group or participant glucose tolerance. The less robust relationship with higher β-cell secretory capacity is likely explained by the small number of non-CF and PS-CF subjects in our sample. A sample containing a larger number of individuals with preserved functional β-cell mass would better define the relationship between MMTT- and GPA-derived measures of β-cell function when β-cell secretory capacity is normal.

In summary, early-phase insulin secretion during a standardized MMTT can provide reliable, and robust measures of β-cell function that agree well with measures of β-cell secretory capacity derived from the more laborious and complex GPA test and may be more favorable for use in large, longitudinal observation or intervention studies across research centers. However, caution should be exercised in extrapolating MMTT ISR results in a largely healthy population in whom β-cell secretory capacity is expected to be normal by GPA, as these MMTT ISR measures may underestimate β-cell functional reserve present in a healthy pancreas.

## Data availability statement

The raw data supporting the conclusions of this article will be made available by the authors, without undue reservation.

## Ethics statement

The studies involving humans were approved by Institutional Review Boards of the University of Pennsylvania and the Children’s Hospital of Philadelphia. The studies were conducted in accordance with the local legislation and institutional requirements. Written informed consent for participation in this study was provided by the participants’ legal guardians/next of kin when appropriate.

## Author contributions

SS: Data curation, Formal Analysis, Writing – original draft, Investigation. DS: Conceptualization, Methodology, Writing – review & editing, Formal Analysis. MK: Data curation, Investigation, Writing – review & editing. DH: Supervision, Writing – review & editing. RR: Conceptualization, Writing – review & editing. MR: Conceptualization, Funding acquisition, Investigation, Methodology, Resources, Supervision, Writing – review & editing. AK: Conceptualization, Funding acquisition, Investigation, Methodology, Resources, Supervision, Writing – review & editing.
